# A one-sheath inverse method in vascular access intervention therapy for hemodialysis patients

**DOI:** 10.1016/j.ijscr.2018.11.067

**Published:** 2018-12-12

**Authors:** Tsuyoshi Takashima, Yasunori Nonaka, Yui Nakashima, Eriko Nonaka, Yuki Ikeda, Makoto Fukuda, Hiroshi Jinnouchi, Shuichi Rikitake, Motoaki Miyazono, Yuji Ikeda

**Affiliations:** a Department of Nephrology, National Hospital Organization, Ureshino Medical Center, 2436 Shimojyukuhei, Uresino-machi, Ureshino, Saga, 843-0393, Japan; b Division of Nephrology, Department of Internal Medicine, Saga University, Faculty of Medicine, 5-1-1 Nabeshima, Saga, 849-8501, Japan

**Keywords:** Hemodialysis, Vascular access intervention therapy (VAIVT), Percutaneous transluminal angioplasty (PTA), Vascular access (VA), Arteriovenous fistula (AVF), One-sheath inverse method

## Abstract

**Introduction:**

Vascular access intervention therapy (VAIVT) is an essential interventional therapy in the field of hemodialysis therapy that allows for the long-term vascular access functionality to be maintained. The venous approach is often performed in VAIVT for arteriovenous fistula. When lesions are present on the upstream and downstream sides from the approach site, it is likely that two sheaths will be inserted from two facing punctures. However, we have adopted a one-sheath inverse method using a guidewire in such cases.

**Case presentation:**

We herein describe the steps of the technique that we have performed and report the successful treatment of a 77-year-old woman who developed arteriovenous fistula failure.

**Discussion:**

To the best of our knowledge, the concrete and detailed technique has not been reported in the English literature to date. The merit of the technique is that it allows VAIVT to be performed using one sheath with one approach site in cases in which lesions are present on the upstream and downstream sides from the approach site. The other benefits include pain reduction, a shortened operation time, and reduced costs. Because vascular access location is usually superficial, the technique can be utilized with relative ease.

**Conclusion:**

A one-sheath inverse method is useful. We hope that the technique will be more widely recognized, allowing the technique to be applied to more cases.

## Highlights

• We describe a one-sheath inverse method in vascular access intervention therapy (VAIVT) for hemodialysis patients.
• It allows VAIVT to be performed using one sheath with one approach site in cases in which lesions are present on the upstream and downstream sides.
• Because vascular access location is usually superficial, the technique can be utilized with relative ease.

## 1 Introduction

Vascular access intervention therapy (VAIVT) is an essential interventional therapy in the field of hemodialysis therapy that allows for the long-term vascular access (VA) functionality to be maintained [[1], [2], [3]]. The venous approach is often performed in VAIVT for arteriovenous fistula (AVF). When lesions are present on the upstream (anastomosis side) and downstream (heart side) sides from the approach site, it is likely that two sheaths will be inserted from two facing punctures. However, we have adopted a one-sheath inverse method using a guidewire in such cases. We herein describe the technique and report the successful treatment of a hemodialysis patient who developed AVF failure.

This work has been reported in line with the SCARE criteria [4].

## 2 Case presentation

**Fig. 1:**
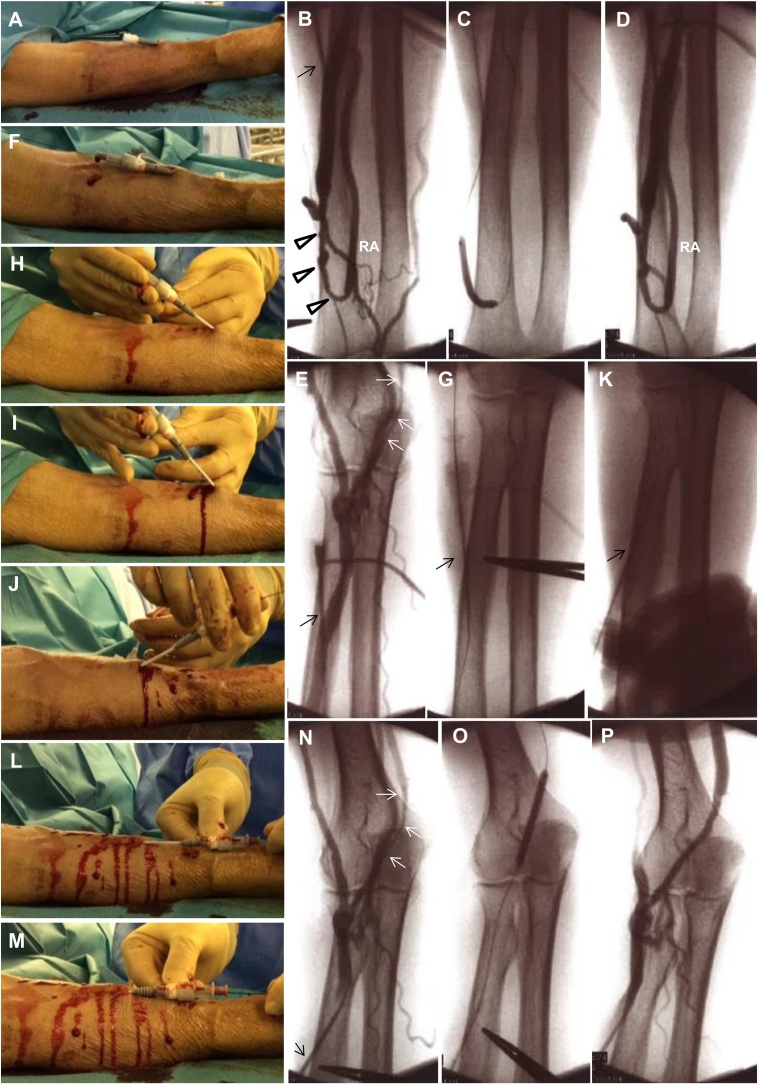
A schematic illustration of the PTA technique and the one-sheath inverse method. (A) Insertion of a sheath in the cephalic vein around the middle of the right forearm toward the anastomosis side by the Seldinger technique. (B) Retrograde angiography showing venous stenosis 0–4 cm from the site of anastomosis (arrowheads), (C) under PTA and (D) post-PTA. Black arrow indicates the puncture point. (E) Antegrade angiography showing stenosis of the median cubital vein (length, approximately 6 cm; white arrows). (F) A dilator and guidewire were threaded into the sheath, and (G) the guidewire was placed in the vein. (H, I) The coaxial dilator/sheath was pulled and gradually stood, and (J) the tip of the dilator/sheath was slightly inverted toward the downstream side. (K) While keeping the state of (J), the distal end of the guidewire was pulled around the tip of the dilator, inverted toward the downstream side, and navigated. (L, M) The coaxial dilator/sheath was carefully reinserted into the vein over the guidewire, and the guidewire and dilator were removed from the sheath. (N) Images showing stenosis of the median cubital vein, (O) under PTA, and (P) post-PTA. In angiography, the upper side reveals the heart side and the lower side reveals the anastomosis side. PTA: percutaneous transluminal angioplasty, RA: radial artery.

A 77-year-old Japanese woman with end-stage renal disease due to chronic glomerulonephritis was introduced to our hospital because of a fourth episode of distal end-to-side radial-cephalic autologous AVF in her right forearm, which had been created 29 months previously. Hemodialysis had been initiated approximately 10 years before the current event, and she had undergone operations related to previous AVFs a total of 21 times: the creation of bilateral radiocephalic AVFs two times and percutaneous transluminal angioplasty (PTA) 19 times. A physical examination revealed a weak vascular murmur and thrill in her right forearm. Preoperative ultrasonography of the right forearm demonstrated venous stenosis located 0–4 cm from the site of anastomosis, and affecting 6 cm of the median cubital vein. Additionally, the diameters of both lesions was <2 mm, and the distal and proximal diameters of the lesions were approximately 4–6 mm. PTA was successfully performed as a salvage operation (Fig. 1A–P).

### 2.1 The VAIVT technique

After disinfecting the patient’s right upper limb, we inserted a 5 Fr × 3 cm sheath (Mosquito Sincere Catheter Introducer, including a guidewire [diameter 0.025 inch × length 50 cm], Boston Scientific Japan K. K., Tokyo, Japan) in the cephalic vein around the middle of her right forearm toward the site of anastomosis by the Seldinger technique [5] (Fig. 1A).

Retrograde angiography from the vein under avascularization revealed venous stenosis 0–4 cm from the site of anastomosis (Fig. 1B). Kyousha^™^ NT Peripheral Guidewire (diameter, 0.018 inch; length, 100 cm; Boston Scientific Japan K. K., Tokyo, Japan) and NSE PTA balloon catheter GDM01 (balloon diameter, 4 mm; length, 4 cm; and rated burst pressure, 18 atm; Nipro corporation, Osaka, Japan) could pass through the lesion. After 2000 units of heparin were administered and allowed to circulate for 5 min, we dilated the lesion several times at 4–18 atm for 30 s, and retrograde angiography showed the improvement of stenosis (Fig. 1C,D).

#### 2.1.1 One-sheath inverse method

We subsequently decided to treat a lesion downstream from the sheath insertion site. First, we detected the stenosis (length, approximately 6 cm) of the median cubital vein by antegrade angiography (Fig. 1E). A dilator and guidewire were threaded into the sheath (Fig. 1F), and the guidewire was placed in the vein (Fig. 1G). Next, the coaxial dilator/sheath was pulled and gradually stood (Fig. 1H,I), and the tip of the dilator/sheath was slightly inverted toward the downstream side (Fig. 1J). While maintaining this state, the distal end of the guidewire was pulled around the tip of the dilator, inverted toward the downstream side, and navigated (Fig. 1K). The coaxial dilator/sheath was carefully reinserted into the vein over the guidewire and the guidewire and dilator were removed from the sheath (Fig. 1L,M).

**Fig. 2:**
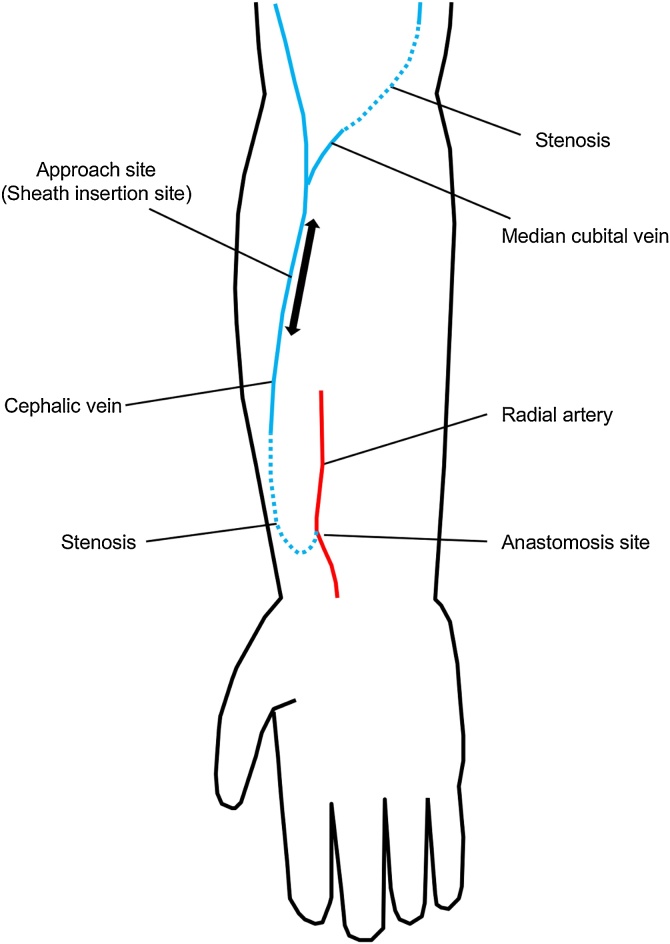
A schematic illustration of the arteriovenous fistula in the patient’s right forearm.

We confirmed the stenosis of the median cubital vein by antegrade angiography again (Fig. 1N). The guidewire and balloon catheter could pass through the lesion; we then dilated the lesion several times at 2–10 atm for 30 s (Fig. 1O). As angiography showed the improvement of stenosis (Fig. 1P), we removed the sheath and finished the procedure. A schematic illustration of her AVF in the right forearm is shown in Fig. 2.

The patient was able to receive hemodialysis therapy uneventfully after PTA.

## 3 Discussion

The Japanese Society of Interventional Radiology guidelines for basic techniques for VAIVT [6] note that, “In cases in which the sheath insertion sites are limited and lesions are present on the upstream and/or downstream sides, it may be difficult to insert two sheaths. In such cases, it can be possible to use a guidewire to insert one sheath using the inverse method.” However, to the best of our knowledge, this concrete and detailed method has not been reported in the English literature. Thus, we introduced the steps of the one-sheath inverse method that we have performed in our department in this article.

As mentioned above, the merit of this technique is that it allows VAIVT to be performed using one sheath with one approach site in cases in which lesions are present on the upstream and downstream sides from the approach site. The other benefits of this technique include pain reduction, a shortened operation time, and reduced costs because only one sheath is used. Incidentally, the performance of PTA using two sheaths in one operation is generally not permitted in Japan.

We described a case with AVF using the technique presented in this article. This technique can be also utilized for arteriovenous graft. VAIVT for arteriovenous graft is most commonly performed using an approach through the graft. Stenosis usually develops at the site of venous anastomosis of the graft and thrombus tends to form within the graft [7,8]. In order to ensure satisfactory inflow and outflow in such cases, sheaths are inserted from opposite sides of the graft on the upstream and downstream sides to perform thrombectomy and PTA. The graft is mainly made of either expanded polytetrafluoroethylene (ePTFE) or polyurethane. This technique is more easily applied than AVF because the wall of the graft is thicker and tougher than an AVF.

The VA location is usually superficial. Thus, the present technique can be utilized with relative ease if operators become accustomed to performing it. However, we should remove the dilator/sheath and perform re-puncture with a needle if we feel strong resistance or catching when inversing and inserting the dilator/sheath. The potential complications of this technique may include bleeding, hematoma, vascular injury, dissection, perforation of the opposite vascular wall, and similar complications. Nevertheless, we have not experienced any serious complications in our department to date.

## 4 Conclusion

The maintenance of VA is a lifeline for hemodialysis patients [9,10]. VA failure is common and can lead to inadequate hemodialysis or VA thrombosis if not identified and treated in a timely fashion. Salvage for VA failure is critical to minimizing catheter use. Therefore, it is important that doctors carefully consider and select various procedures to preserve patients’ VA whenever possible. We hope that, with the introduction of the above guidelines, this present technique will be more widely recognized, allowing the technique to be applied to more cases.

## Conflict of interest

The authors declare no conflict of interest.

## Funding

This research did not receive any specific grant(s) from funding agencies in the public, commercial, or not-for-profit sectors.

## Ethical approval

No Institutional Review Board is required for publication of a case report at our institution.

## Consent

Informed consent was obtained from the patient for publication of this case report and any accompanying images.

## Author contribution

Nonaka Y made the surgical intervention. Takashima T and Nakashima Y participated to the surgical intervention. Takashima T, Nonaka E, Ikeda Yuki, Fukuda M, Jinnouchi H, Rikitake S, Miyazono M contributed in the collection of the data. Takashima T drafted and edited the manuscript. Ikeda Yuji gave final approval of the manuscript.

## Registration of research studies

The paper is not a research study.

## Guarantor

Takashima T.

## Provenance and peer review

Not commissioned, externally peer-reviewed
